# Cochlear Implant Decisions in South Africa: Parental Views, Barriers, and Influences

**DOI:** 10.3390/healthcare13070787

**Published:** 2025-04-01

**Authors:** Katijah Khoza-Shangase, Jasmine Bent

**Affiliations:** Department of Audiology, School of Human & Community Development, University of the Witwatersrand, Johannesburg 2050, South Africa; jasminebent51@gmail.com

**Keywords:** cochlear implants, parental decision-making, South Africa, socio-economic barriers, hearing loss, Deaf identity, Q-methodology, mixed methods, healthcare accessibility, stigma

## Abstract

**Background**: Cochlear implants (CIs) have become a widely used intervention for Deaf and hard-of-hearing (DHH) children, particularly for developing spoken language. However, parental decision-making regarding CIs is influenced by a range of factors, including socio-economic status, healthcare accessibility, cultural beliefs, and societal attitudes. While extensive research on parental perceptions of CIs exists in high-income countries (HICs), there is limited research on these perspectives in low- and middle-income countries (LMICs), like South Africa, where disparities in healthcare access significantly impact CI uptake. **Objectives**: This study aimed to explore the views and perceptions of South African parents regarding CIs for their DHH children, with a specific focus on how financial, cultural, and informational barriers influence decision-making. **Methods**: A mixed-methods approach was used, combining Q-methodology for quantitative data and thematic analysis for qualitative insights. Nine parents of DHH children participated. The Q-set survey ranked parental attitudes toward CI risks, benefits, and accessibility, while semi-structured interviews provided deeper insights into decision-making processes. Factor analysis grouped participants into clusters based on their perceptions, and qualitative data were analysed using a thematic framework approach. **Results**: Findings revealed two distinct parental clusters: (a) Cluster 1 parents viewed CIs as essential for speech development and strongly supported implantation, and (b) Cluster 2 parents recognized CI benefits but emphasized that outcomes vary based on individual circumstances. Three overarching themes emerged from thematic analysis: (1) financial barriers restricting CI access, (2) parental reliance on medical professionals for decision-making, and (3) persistent stigma and cultural beliefs influencing CI perceptions. **Conclusions**: This study highlights critical barriers to CI access in South Africa, including socio-economic inequities, limited healthcare infrastructure, and persistent stigma. While parents largely recognized the benefits of CIs, their decisions were shaped by financial constraints and concerns about Deaf identity and societal acceptance. This study calls for the expansion of publicly funded CI programmes, the development of culturally tailored parental counselling protocols, and targeted public awareness campaigns to reduce stigma surrounding hearing restoration devices. These interventions can help mitigate financial and cultural barriers to CI adoption in South Africa.

## 1. Introduction

Hearing loss is one of the most common congenital conditions worldwide [1,2]. Being Deaf or hard-of-hearing (DHH) does not equate to an inability to communicate, as various communication modes exist, including those facilitated by hearing restoration devices like cochlear implants (CIs) [3,4]. Since their introduction in the late 20th century, cochlear implants have become a widely used intervention among parents seeking to develop their child’s spoken language, particularly in high income countries (HICs) [5,6,7]. However, despite growing CI adoption globally, access and uptake remain limited, particularly in low- and middle-income countries (LMICs) like South Africa, where factors such as financial constraints, parental choice, and healthcare limitations impact decision-making [8,9,10,11,12,13]. Parental decision-making regarding cochlear implantation is shaped by a complex interplay of attitudes, perceptions, and beliefs, which are further influenced by financial constraints, access to information, and cultural perspectives. Parents often rely on healthcare professionals for guidance, but access to information is not always equitable, particularly for lower-income families. Additionally, the high cost of CI implantation in South Africa creates financial barriers that impact whether parents view this intervention as a viable option for their child. These factors highlight the importance of understanding parental perspectives within a socio-economic and healthcare access framework. In this study, ’attitudes’ refers to parents’ overall disposition towards cochlear implants, ’perceptions’ encompass how they interpret the potential risks and benefits of CIs, and ’beliefs’ reflect the underlying convictions that guide their decisions. These factors interact with socio-economic realities, cultural perspectives, and access to healthcare, ultimately influencing parental decision-making around cochlear implantation for their child.

In South Africa, the challenges of CI access are compounded by disparities between public and private healthcare [14]. The majority of South Africans rely on the public sector, which faces financial and resource limitations, affecting the availability of interventions like CIs [9,15]. Geographic disparities further exacerbate the issue, with rural communities experiencing significantly lower access to CI programmes [9]. While CIs have been associated with better speech development in children with no comorbidities [1,7,16], they remain a controversial intervention. Some members of the Deaf community view CIs as a threat to Deaf culture, arguing that sign language should be prioritized to preserve identity and linguistic heritage [5,17,18]. Others contend that spoken language is essential for social integration in a predominantly hearing world [5]. This ethical debate raises the complexity of CI decision-making, particularly for parents, who must navigate medical recommendations, financial constraints, and cultural considerations [5,19].

While extensive research exists on parental attitudes toward CIs in HICs [5,20], studies in LMICs, particularly in South Africa, remain scarce. Furthermore, no existing study has systematically examined how socio-economic constraints, healthcare disparities, and cultural perspectives collectively shape CI decision-making in South Africa. South African studies, such as those by Kanji, Mirkin, and Casoojee [21], Brewis et al. [7], Bhamjee et al. [8], and Moroe and Kathrada [22], have explored various aspects of CI decision-making, including general parental experiences, emotional responses, and perceived health-related quality of life, or emotional responses, rather than systematically analysing how socio-economic status, financial barriers, and cultural beliefs shape decision-making. Additionally, most prior studies did not employ Q-methodology, which allows for the identification of distinct parental viewpoints rather than generalized responses. Furthermore, earlier studies did not incorporate a range of income levels, limiting the ability to analyz how financial constraints impact parental attitudes toward CI accessibility and affordability.

This study extends prior research by explicitly examining how socio-economic status, financial constraints, and cultural beliefs influence parental decision-making regarding CIs in South Africa. Unlike previous studies that primarily focused on general attitudes toward CIs, this study investigates the specific factors that shape parents’ choices—including financial affordability, trust in medical professionals, and societal stigma—which remain underexplored in the South African context. Given the disparities in healthcare access and the cultural significance of Deaf identity, we expect to find variability in parental attitudes toward CIs. Specifically, we anticipate that: (1) parents from lower-income backgrounds may perceive CIs as financially unattainable, affecting their willingness to consider implantation; (2) parents with limited exposure to the Deaf community may be more inclined to view CIs as the ‘best option’ for communication development; and (3) medical professionals play a central role in guiding parental decisions, but trust in their recommendations may vary depending on socio-economic status and access to healthcare. By identifying key themes and contrasting parental viewpoints, this study aims to contribute actionable insights for improving CI accessibility, healthcare communication, and parental support systems in South Africa. By incorporating both statistical clustering and qualitative inquiry, this research offers a uniquely structured yet in-depth understanding of parental viewpoints on CI adoption.

Parental perspectives are critical in shaping early intervention strategies for DHH children. Parents serve as primary decision-makers in choosing communication modes and assistive devices, decisions that have long-term implications for speech, language, and cognitive development [7,21,22]. Given the increasing prevalence of CI programmes in South Africa [9], it is essential to understand the factors influencing parental decisions—such as financial constraints, information accessibility, and social stigma—to ensure that intervention programmes are culturally and contextually relevant.

Currently, South Africa has 12 CI programmes operating across five of its nine provinces, with most services concentrated in private healthcare settings, which cater to only 14% of the population [9,23,24]. The limited accessibility of CIs for the majority of South Africans, particularly those in rural areas, underscores the need for research that informs equitable policy and intervention strategies [10,23]. This study has two primary objectives. First, it explores parents’ perceptions of the risks, benefits, and outcomes of CIs, providing insight into how South African parents assess the advantages and limitations of CIs within their socio-economic and cultural contexts. Second, it examines the key factors influencing parents’ decisions regarding CIs, highlighting the broader implementation challenges faced by CI programmes in South Africa. By identifying the barriers and motivators shaping parental decision-making, this study aims to contribute to policies and intervention strategies that improve CI accessibility, particularly for underserved communities.

As far as novelty is concerned, this research contributes new knowledge by systematically analysing how socio-economic and cultural factors influence parental perspectives on CIs. While previous studies [7,21] have explored parental views, they have not extensively examined the intersection of financial constraints, healthcare access, and cultural beliefs in shaping decision-making. Through Q-methodology and thematic analysis, this study provides a structured yet in-depth exploration of parental viewpoints. Although the sample was not stratified by income level or cultural background, emerging themes from the data reflect the role of these factors in shaping parental decision-making.

Ultimately, this study aims to explore the views and perceptions of South African parents regarding CIs for their DHH children. By examining parental attitudes toward the risks, benefits, and outcomes of CIs, as well as the socio-economic and cultural factors influencing decision-making, this research seeks to contribute to the development of more contextually relevant early intervention strategies. The specific objectives of this study are to explore parents’ perceptions of the risks, benefits, and outcomes of CIs; and to investigate the key factors influencing parental decisions regarding whether to pursue cochlear implantation for their child. By identifying these factors, the study will offer insights into how healthcare systems and policies can better support parents in making informed decisions about CIs, particularly within the resource-limited South African context.

## 2. Materials and Methods

### 2.1. Study Design

This study employed a mixed-methods approach, incorporating Q-methodology for quantitative analysis and thematic analysis for qualitative insights to explore the diversity of parental perspectives on CIs. Q-methodology was employed to systematically categorize parents into distinct attitudinal clusters based on their responses to a structured Q-set, while inductive thematic analysis was applied to semi-structured interview data to identify recurring themes in parental decision-making. The rankings were analysed using factor analysis, employing Pearson’s correlation coefficient and Horst’s centroid method to identify clusters of similar parental perceptions [25,26,27]. The semi-structured interviews enabled participants to elaborate on their responses, offering deeper insights into decision-making processes, attitudes, and socio-cultural influences. The qualitative data were analysed using inductive thematic analysis, following Braun and Clarke’s [28] guidelines.

Q-methodology was specifically chosen over traditional quantitative techniques such as standardized surveys or regression analysis because it allows for the systematic identification of shared perspectives rather than relying on pre-determined variables. Unlike surveys, which aggregate responses into general trends, Q-methodology reveals distinct attitudinal clusters, highlighting areas of both consensus and divergence among participants. This is particularly valuable for understanding subjective decision-making processes, such as the complex interplay of socio-economic constraints, cultural beliefs, and trust in medical professionals that influence CI adoption. Furthermore, standard survey methods often assume independence of responses, while Q-methodology captures relational viewpoints, grouping participants based on shared patterns of thinking. Given the small sample size in this study, Q-methodology was a more appropriate choice than regression analysis, which typically requires larger datasets to generate statistically significant predictors. By combining Q-methodology with thematic analysis, this study ensures a structured yet in-depth exploration of parental perspectives on CIs.

The mixed-methods approach adopted in this study ensured a comprehensive understanding of both patterned attitudes and nuanced qualitative insights on CIs within the South African context.

### 2.2. Study Setting

The study was conducted in South Africa, where access to CIs is highly variable due to financial, healthcare, and geographic disparities [10,15]. Participants were recruited from The Children’s Communication Centre in Johannesburg as well as online platforms to ensure broader representation across different regions. Online recruitment has the advantage of reaching a broad geographic range of participants, but it can also introduce accessibility biases, particularly for parents from lower-income or rural backgrounds who may have limited internet access. To mitigate this, recruitment was conducted across multiple digital platforms, including social media, audiology-related forums, and community-based parent support groups. Additionally, outreach was extended to audiology clinics and schools serving DHH children, where parents were invited to participate regardless of their digital access. However, despite these efforts, parents without internet connectivity or regular engagement with healthcare services may still be underrepresented in the sample.

### 2.3. Participants

#### 2.3.1. Inclusion Criteria

While this study emphasizes socio-economic and cultural influences, stratifying the sample by income or cultural background was not feasible due to the limited pool of eligible participants and challenges in recruitment. However, the study still captured diverse financial and educational backgrounds. Educational attainment also varied, with participants holding either high school diplomas or bachelor’s degrees. This variation allowed for differing perspectives on affordability, accessibility of healthcare information, and trust in medical professionals. The study, therefore, provides valuable insights into socio-economic influences on CI decision-making, even without formal stratification. Future research should aim for a larger, stratified sample to explore more granular differences across cultural, linguistic, and economic subgroups in South Africa.

Participants had to meet the following criteria:

- Parents/legal guardians of DHH children.
- South African citizens or permanent residents.

#### 2.3.2. Exclusion Criteria

- Individuals under 18 years of age.
- Parents/guardians unable to provide informed consent.

### 2.4. Sampling Technique

A non-probability, convenience sampling and voluntary response sampling strategy was used [29]. This approach ensured the inclusion of parents who had direct experience with CIs while also considering accessibility constraints. Although the sample size was relatively small (n = 9), Q-methodology prioritises capturing diverse perspectives rather than achieving statistical generalizability. This approach is widely used in exploratory research to identify distinct viewpoints within a population, even with small samples [26].

Despite the lack of formal stratification, this study still incorporated a range of socio-economic backgrounds, with participants spanning lower-class to upper-middle-class income levels. The diversity in income and education levels allowed for meaningful exploration of how financial stability, healthcare access, and information-seeking behaviors influence CI decision-making. Given the niche population of parents making decisions about cochlear implantation in South Africa, prioritizing depth over breadth was necessary. The chosen methodology ensured a rich, qualitative understanding of key parental concerns, financial constraints, and healthcare perceptions, providing valuable insights despite the modest sample size.

Efforts were made to recruit participants from both urban and rural areas through online platforms, social media, and outreach to audiology clinics and schools serving DHH children. However, the majority of respondents who volunteered for participation were from urban or peri-urban areas, which may reflect greater access to online recruitment platforms and higher engagement with audiology services in these regions. While parents from rural areas were not explicitly excluded, limited digital access and healthcare outreach barriers may have contributed to their underrepresentation in the sample.

### 2.5. Data Collection

#### 2.5.1. Instruments

The study employed:

1. Q-set Survey: A list of 27 statements (Appendix A) related to CIs, rated by participants using an unforced distribution, allowing them to agree or disagree with as many statements as they wished [25,26,30].
2. Semi-structured Interviews: Conducted via phone or in-person, recorded, and transcribed for thematic analysis.

The survey was initially hosted on Q-sortware (http://www.qsortware.net) but later migrated to Survey Planet due to accessibility issues identified in the pilot study. Survey Planet was chosen for its mobile accessibility, making it more suitable within the South African context, where smartphones are more commonly available than laptops or desktops.

The Q-set and interview questions were developed using peer-reviewed research, literature reviews, and mass media [5,7,19,20,21,31,32,33,34]; as well as consultation with audiology experts to ensure relevance to South African socio-economic and cultural factors [15]. Rather than explicitly asking about financial or cultural barriers, statements such as “CI surgery is unaffordable for most South Africans” and “People with CIs are still a part of the Deaf community” were used to capture indirect socio-economic and cultural influences on parental perspectives.

#### 2.5.2. Procedure

Ethical clearance was obtained from the University of the Witwatersrand’s Human Research Ethics Committee (Non-medical) (Protocol number: STA_2023_15). Then a pilot study was conducted with two participants to refine the survey and interview questions [35]. Following the pilot study, several refinements were made to the survey and interview questions to enhance clarity and relevance. Participants reported that some statements in the Q-set were ambiguous, leading to modifications in wording to ensure clearer distinctions between response options. Additionally, minor adjustments were made to the phrasing of interview questions to encourage more open-ended and elaborative responses, particularly regarding socio-economic influences on CI decision-making. The overall study design remained unchanged, as the pilot confirmed the feasibility of the mixed-methods approach. However, technical aspects of data collection were adjusted—for instance, the original Q-set was hosted on Q-sortware, but pilot participants found the platform difficult to navigate. As a result, the survey was migrated to SurveyPlanet (https://surveyplanet.com), which was more user-friendly and mobile-accessible, ensuring broader participant engagement.

Participants completed the online Q-sort survey, followed by optional semi-structured interviews. Survey data were analysed using factor analysis, and interviews were thematically analysed.

### 2.6. Data Analysis

#### 2.6.1. Quantitative Analysis (Q-Methodology)

This involved factor analysis using Ken-Q Analysis software (Version 2.0.1) to group participants into clusters based on response patterns [26,30]. Pearson’s correlation coefficient and Horst’s centroid method were applied to identify similarities among participants [32,36].

#### 2.6.2. Qualitative Analysis (Thematic Analysis)

Qualitative data were analysed using Braun and Clarke’s [28] six-step thematic analysis framework, ensuring systematic identification of themes across parental narratives. This involved familiarization with data, generating initial codes, searching for themes, reviewing themes, defining themes, and then producing the final report. Member checking ensured that participants reviewed their responses for accuracy.

### 2.7. Ethical Considerations

This study adhered to ethical guidelines outlined in The Belmont Report [37] and the HPCSA General Ethical Guidelines for the Healthcare Professions [38]. For anonymity and confidentiality, the online survey was fully anonymous with no identifying data collected; while for interviews, confidentiality was ensured through pseudonyms and data anonymization. Participant welfare was assured with the distress protocol that was put in place [39,40]. Given the potentially emotionally sensitive nature of discussing cochlear implantation, a distress protocol was established to ensure participant well-being. Interviewers were trained to recognize signs of emotional distress, such as prolonged pauses, difficulty speaking, or expressions of distress (e.g., crying or frustration). If a participant exhibited signs of distress, the interviewer paused the session, offered reassurance and the option to take a break, and allowed the participant to either continue or withdraw from the study without any consequences. Participants were also provided with referral information for psychological and emotional support services, including access to counselling services at audiology clinics and national mental health helplines. No participants withdrew due to distress, but one participant requested a brief pause during the interview, which was accommodated before proceeding.

Participants could withdraw at any stage without penalty. All participants were provided with a detailed consent form outlining the study’s purpose, procedures, potential risks, and their right to withdraw at any time. Written informed consent was obtained from all participants before their involvement in the study, ensuring ethical compliance with the University of the Witwatersrand’s Human Research Ethics Committee guidelines (Protocol Number: STA_2023_15).

### 2.8. Rigor and Trustworthiness

This study applied Lincoln and Guba’s [41] criteria. For credibility, member checking was used to verify findings, and peer debriefing reduced researcher bias. For transferability, thick descriptions enabled broader application of findings. For dependability, an audit trail documented all methodological decisions. Lastly, for confirmability, reflexive journaling ensured transparency, and triangulation between Q-methodology and thematic analysis improved reliability.

When it comes to quantitative validity and reliability, content, face, and Q-sorting validity were tested through a pilot study [42]. The Q-set was based on thorough literature review and exclusion of jargon to enhance clarity.

### 2.9. Data Management

All digital data (survey responses, interview transcripts) were securely stored on password-protected servers. Audio recordings were transcribed and deleted post-analysis. Data were de-identified for confidentiality.

## 3. Results

### 3.1. Participants’ Profile

Table 1 provides an overview of the demographic characteristics of the participants’ children. Key findings include:

- School Attendance: Seven out of nine (n = 7) participants reported that their children do not attend a school for the Deaf.
- Use of Hearing Assistive Devices: Seven out of nine (n = 7) parents reported that their children use hearing aids, but only four (n = 4) reported consistent use.
- Modes of Communication:
  ○ Three (n = 3) participants reported that their children primarily use speech.
  ○ Three (n = 3) reported that their children primarily use gestures.
  ○ Two (n = 2) reported total communication (combination of speech, gestures, and sign language).
  ○ One (n = 1) participant reported that their child uses sign language as the primary mode of communication.
- Income Range: participants represented a diverse socio-economic background, with one (n = 1) from the lower-class income group (ZAR 3500–R8000), four (n = 4) from the lower-middle class (ZAR 8000–ZAR 22,000), two (n = 2) from the middle class (ZAR 22,000–ZAR 40,000), and two (n = 2) from the upper-middle class (ZAR 40,000–ZAR 75,000).
- Educational Qualification: participants also had varying educational backgrounds, with one (n = 1) having completed grade 9, two (n = 2) holding a high school diploma, and six (n = 6) having attained a bachelor’s degree.

These demographic findings highlight that most of the participants’ children do not have access to Deaf education or South African Sign Language (SASL) and rely on hearing aids or spoken language, which may influence their parents’ perceptions of CIs. Additionally, the range of income levels and educational qualifications provides insight into how socio-economic status may affect CI decision-making, particularly in terms of access to healthcare information, perceived affordability, and trust in medical professionals.

### 3.2. Clustering of Parental Perceptions

Participants were divided into two clusters based on their correlation matrix scores (Table 2). The clustering process involved generating a correlation matrix that assessed the degree of similarity between participants’ Q-sort rankings. The Pearson’s correlation coefficient was used to measure how closely each participant’s response pattern aligned with others. To extract distinct clusters of viewpoints, factor analysis was performed using Ken-Q Analysis software, applying Horst’s centroid method to identify common response patterns. Factor loadings were then calculated to determine the degree to which each participant’s responses aligned with a given cluster. A threshold of 0.40 or higher was used to assign participants to a cluster, ensuring that only participants with strong alignment to a specific factor were grouped together. Those who did not meet this threshold were considered non-significant loaders and were not assigned to any specific cluster:

- Cluster 1 (n = 3): Participants 2, 3, and 4
- Cluster 2 (n = 6): Participants 1, 5, 6, 7, 8, and 9

Cluster 1 participants strongly endorsed cochlear implantation, prioritizing speech development, while Cluster 2 expressed conditional support, emphasizing the child’s individual circumstances and the potential limitations of CIs. These clusters serve as a basis for analyzing differences in parental perceptions in the next sections.

Cluster 1 parents, who strongly endorsed CIs for speech development, tended to have children enrolled in mainstream education and expressed concerns about stigma and societal barriers to alternative communication methods. Conversely, Cluster 2 parents, while acknowledging CI benefits, emphasized individualized decision-making and were more likely to question the universal applicability of CIs. These differences highlight the intersection of educational choices, societal expectations, and financial realities in shaping CI perceptions.

### 3.3. Exploring Parents’ Perceptions of the Risks, Benefits, and Outcomes of CIs

#### 3.3.1. Perceived Risks of CI Surgery

**Figure 1:**
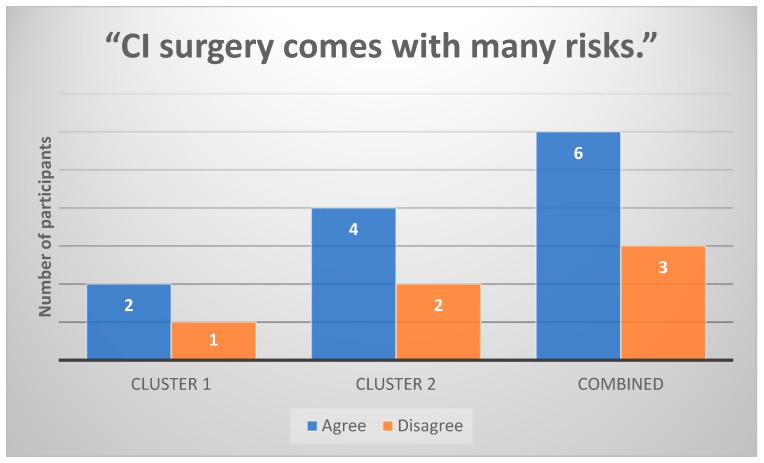
Participants’ responses to the statement “CI surgery comes with many risks” (n = 9).

Six out of nine (n = 6) participants agreed that CI surgery involves risks. The statement “CI surgery comes with many risks” was the second-most agreed-upon across all participants (Figure 1).

#### 3.3.2. Financial Barriers to CIs

**Figure 2:**
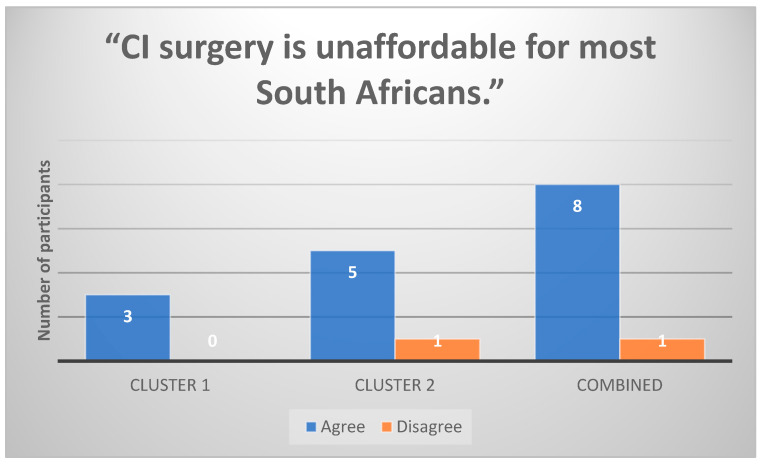
Participants’ responses to the statement “CI surgery is unaffordable for most South Africans” (n = 9).

All Cluster 1 participants (n = 3) strongly agreed that CI surgery is unaffordable for most South Africans (Figure 2). Financial barriers were a common theme influencing decision-making, as parents expressed concerns about medical aid limitations and out-of-pocket expenses.

#### 3.3.3. Belief in CIs as the Best Option for Speech Development

**Figure 3:**
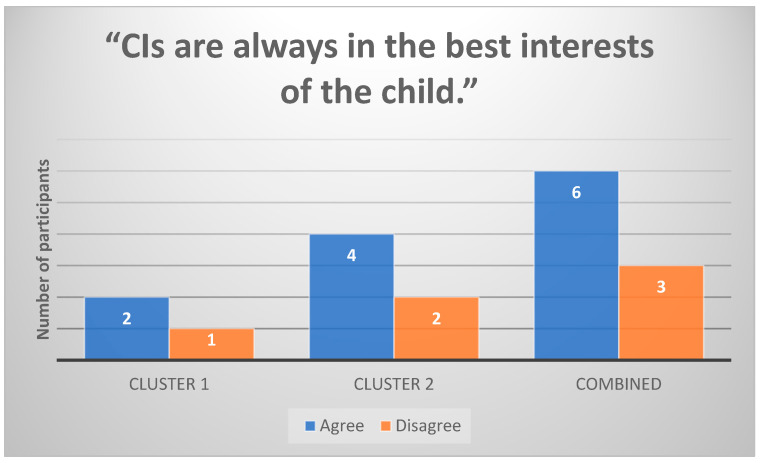
Participants’ responses to the statement “CIs are always in the best interests of the child” (n = 9).

Two out of three (n = 2) Cluster 1 participants strongly believed that CIs are always in the best interests of the child (Figure 3). In contrast, Cluster 2 participants (n = 6) were more hesitant about this claim and emphasized that CI benefits vary depending on the child’s individual needs.

#### 3.3.4. Identity and Social Integration Concerns

**Figure 4:**
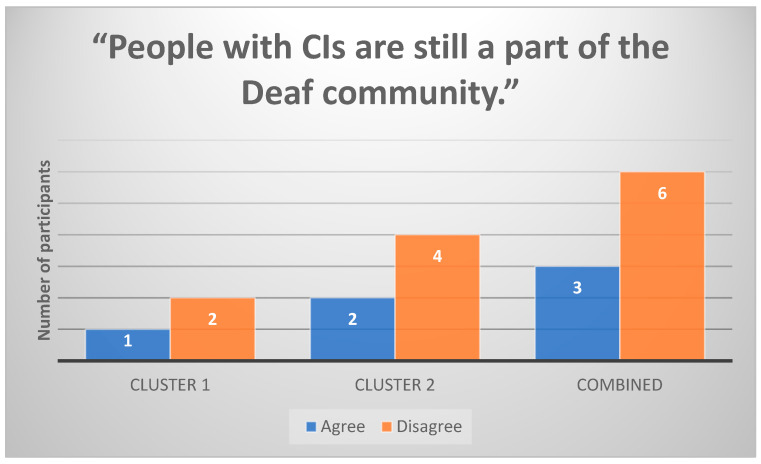
Participants’ responses to the statement “People with CIs are still a part of the Deaf community” (n = 9).

Cluster 1 participants were less likely to view CI users as part of the Deaf community (Figure 4). However, some Cluster 2 participants (n = 6) acknowledged that CIs do not fully replace Deaf identity, emphasizing the importance of language and cultural identity alongside medical intervention.

### 3.4. Thematic Analysis of Parental Views on CIs

Under this objective, three key themes emerged:

Theme 1: *CIs Are Perceived as More Effective for Speech Development*

Cluster 1 participants expressed a strong belief that CIs provide superior speech outcomes compared to hearing aids.

*“If we could have done the implant, yes, it would have been better for [my child]”*

Anne (P2)

*“They will develop good speech [with the implant]. Their speech would develop faster and better than if they are not having that correction”*

Solag (P3)

Theme 2: *CIs Benefits Depend on Individual Circumstances*

Cluster 2 participants acknowledged potential CI benefits but emphasized that outcomes depend on the child’s specific condition, learning environment, and support system.

*“I think it is a sensitive topic because every individual—some of them will need cochlear implants and others hearing aids”*

LSG (P5)

*“The deaf lady with a cochlear implant who I know is definitely very satisfied with her implant… But I think in some cases a cochlear implant won’t be effective”.*

Rolf (P1)

Theme 3: *Stigma and Societal Perceptions of CIs*

Participants from both clusters noted stigma surrounding hearing assistive devices and CIs, especially within schools and workplaces. Some referenced cultural beliefs that impact CI acceptance.

*“Our society, I think there is some element of discrimination. Because with the hearing aid there are certain jobs that they might not be accepted for”.*

Solag (P3)

*“Part of it is parents have certain superstitions about certain conditions… You have to get the children to realize that it’s normal”.*

Sally (P6)

### 3.5. Investigating Influencing Factors Behind Parental CI Decisions

Similarly, under this objective, three key themes emerged:

Theme 1: *Reliance on Medical Practitioners*

Seven out of nine (n = 7) participants cited medical professionals as their primary source of CI information. However, one participant (P2) expressed scepticism about whether medical professionals provide complete and unbiased information.

*“I trusted the people that were helping me with my child. I trusted that they knew better than me”.*

LSG (P5)

*“Most medical practitioners will not come out telling you the truth about all the side effects. One has to be very careful”.*

Anne (P2)

Moreover, three participants (n = 3) actively sought second opinions from other medical professionals, and two participants (n = 2) engaged in independent research through online sources or support groups. While some parents expressed complete trust in their primary healthcare provider’s recommendations, others acknowledged a desire for additional perspectives before making a final decision. For example, one participant stated:

*“I did not just go with the first advice; I consulted different doctors to see if they all said the same thing”.*

(P2)

Conversely, others felt overwhelmed by conflicting information, leading them to rely solely on their audiologist or ENT specialist for guidance. A few participants also mentioned speaking with other parents of children with CIs to gain insights from lived experiences, though these informal discussions were secondary to medical consultations.

Theme 2: *Lack of Knowledge and Overwhelming Decision-Making Process*

Several participants reported feeling unprepared when making decisions about their child’s hearing intervention. Parents described difficulty finding reliable information and navigating different medical opinions.

*“I think it’s hard when somebody has a child that is different… because you don’t even know who to go to, who to trust”.*

Sally (P6)

*“Everything was new to us. South Africans need to be more educated about this business and hard-of-hearing people”.*

LSG (P5)

Theme 3: *Financial Constraints and Personal Circumstances*

Several parents expressed that financial constraints influenced their ability to pursue CIs. Some noted that even medical aid support was insufficient to cover the full costs of implantation and rehabilitation.

*“Now it’s a financial thing… What can we afford? What is the medical aid willing to pay?”*

Sally (P6)

*“In her case, a cochlear implant would not have benefitted her”.*

Rolf (P1)

Overall, this study’s key findings were that, firstly, parents’ perceptions of CI risks and benefits varied by cluster, with Cluster 1 participants (n = 3) supporting CIs for speech development, while Cluster 2 participants (n = 6) emphasized individual variability. Secondly, financial barriers and stigma emerged as key obstacles to CI uptake. Thirdly, parents heavily relied on medical professionals but lacked broader knowledge on CI options. Lastly, decision-making was influenced by both practical constraints (e.g., finances) and personal beliefs about disability and identity.

## 4. Discussion

This study provides new insights into how socio-economic and cultural factors intersect with parental perceptions of CIs in the South African context. While previous studies have examined parental decision-making regarding CIs (e.g., [21,22]), this study uniquely applies Q-methodology to categorize distinct viewpoints and supplements these findings with qualitative data, offering a deeper understanding of how financial constraints, cultural beliefs, and healthcare access shape parental choices. By contextualizing these findings within South Africa’s healthcare landscape, this study highlights specific barriers that contribute to inequitable access to CIs. The mixed-methods approach facilitated an in-depth exploration of the factors influencing parental decision-making, providing insights into both statistical clustering of viewpoints and the nuanced experiences of parents navigating the CI process.

The demographic profile of participants’ children revealed that mainstream schooling was preferred over Deaf education, with seven out of nine participants (n = 7) reporting that their children do not attend a school for the Deaf. This trend aligns with the global and South African literature, which suggests that hearing parents often prioritize spoken language and mainstream education over Deaf schooling and sign language acquisition [5,43]. The preference for mainstream education reflects a desire for social and economic integration, as parents may perceive spoken language skills as critical for future success [44]. Similarly, most parents reported relying on hearing aids rather than sign language for communication. Despite seven out of nine (n = 7) children using hearing aids, only four (n = 4) used them consistently, reflecting findings by Chang [31] and Jefferis [45] that comfort, stigma, and effectiveness influence device usage. The low prevalence of sign language usage (n = 1) further reinforces previous studies showing that hearing parents often deprioritize sign language acquisition in favour of oral communication approaches [5,20]. The implications of these findings are significant. The limited exposure to sign language and Deaf education among the children in this study suggests that many parents operate within a framework that prioritizes spoken language over Deaf cultural identity. This orientation is likely to shape parental attitudes toward cochlear implantation, with parents viewing CIs as a tool for facilitating speech development and integration into the hearing world.

A key finding in this study was the perception that CIs are more effective for speech development compared to other hearing assistive devices. This view was particularly evident in Cluster 1 participants, who strongly endorsed early implantation for optimal speech and language outcomes. Their belief aligns with literature demonstrating that early cochlear implantation can lead to significant improvements in spoken language acquisition for DHH children [1,7,16]. However, Cluster 2 participants expressed more nuanced views, emphasizing that CI benefits vary based on individual circumstances such as the child’s specific hearing loss, learning environment, and available support systems. This perspective reflects a growing recognition in CI research that while CIs can be beneficial, outcomes are not uniform and depend on multiple factors [16]. These differing views have implications for parental counselling and CI candidacy evaluation. While medical professionals often emphasize the benefits of early implantation, the perspectives of Cluster 2 participants suggest that more personalized, context-sensitive discussions are needed to ensure that parents make informed choices based on their child’s unique needs rather than a generalized expectation of CI success.

A consistent finding across both clusters was that financial constraints significantly impact CI decision-making. Participants widely acknowledged that CIs are unaffordable for most South Africans, particularly those reliant on public healthcare. This aligns with research showing that South Africa’s dual healthcare system disproportionately limits access to specialized interventions for lower-income populations [10]. The high cost of CI surgery, device maintenance, and rehabilitation services creates systemic barriers, preventing many eligible children from receiving implantation [15]. These findings highlight the need to critically evaluate the accessibility of CI programmes within the public sector, and to increase the number of programmes that currently exist. The socio-economic disparities identified in this study suggest that CI uptake may remain largely confined to wealthier families with access to private healthcare, exacerbating existing inequalities in early hearing intervention and speech development outcomes. Given that CIs are predominantly available in private healthcare settings, integrating CI funding into South Africa’s NHI scheme could be a crucial step toward reducing disparities. Policy interventions aimed at increasing public CI programmes would improve access for low-income families, particularly in rural and under-resourced communities

Parents in this study relied heavily on medical professionals for guidance in deciding whether to pursue cochlear implantation. This reliance is well-documented in the literature, where audiologists, otolaryngologists, and speech therapists are primary sources of information for parents considering CIs [6,12,46]. However, while most parents trusted healthcare professionals, one participant expressed scepticism about the completeness and transparency of medical advice regarding CI risks. This scepticism reflects concerns raised in previous studies, where parents have reported feeling pressured or inadequately informed about potential long-term challenges associated with CIs [47]. While most participants relied primarily on medical professionals, some expressed a need for additional verification before making a final decision. Seeking second opinions was more common among parents who had concerns about potential risks, whereas those who felt overwhelmed by information tended to rely exclusively on their primary healthcare provider. This aligns with previous findings that trust in medical professionals significantly influences parental confidence in healthcare decisions [12]. Additionally, online research and peer discussions played a minor but noteworthy role in supplementing medical advice. Parents who consulted other CI families reported feeling more reassured about post-implantation experiences, whereas those who used online sources described encountering conflicting information that sometimes added uncertainty to their decision-making process. These findings highlight the dual role of medical professionals as both trusted authorities and gatekeepers of information, suggesting that ensuring clear, transparent, and comprehensive communication could help parents feel more empowered in their choices.

The implications of this finding underscore the need for balanced, transparent communication in CI counselling. Ensuring that parents receive unbiased, comprehensive information about both the benefits and limitations of CIs is critical to fostering informed decision-making. While parents in this study relied heavily on medical practitioners, concerns about the transparency of information suggest a need for standardized, unbiased counselling protocols. Providing parents with balanced perspectives—including both potential benefits and limitations of CIs—could foster more informed decision-making and mitigate any undue influence from external pressures.

Stigma surrounding hearing loss and assistive devices emerged as a salient theme across both clusters. Participants reported concerns about social exclusion, discrimination in schools and workplaces, and cultural beliefs influencing perceptions of disability. The finding that hearing loss stigma remains prevalent in South Africa aligns with previous studies that suggest superstitions and negative societal attitudes toward disabilities persist [48,49,50]. Some participants also noted that assistive devices, including CIs, are not always socially accepted, which can contribute to self-consciousness or reluctance to use them. The implications here suggest that stigma could be a major factor discouraging some parents from choosing CIs for their children, even when they acknowledge potential benefits. Efforts to address negative perceptions of hearing assistive devices may be necessary to ensure that stigma does not act as a barrier to intervention uptake.

These findings align with theoretical models of health decision-making that emphasize the role of financial, cultural, and informational accessibility in shaping parental choices. The reliance on medical professionals suggests that South African parents may experience decision fatigue when faced with conflicting messages about CI benefits and risks. Future policy interventions should not only focus on increasing access to CI surgery but also on improving decision-making support through evidence-based counselling and culturally responsive communication strategies.

While this study provides valuable insights into parental perspectives on CIs in South Africa, several limitations must be acknowledged. First, the small sample size which included nine participants limits the generalizability of findings to the broader population. However, Q-methodology does not rely on large sample sizes for validity, as its goal is to identify clusters of viewpoints rather than to generate population-wide generalizations. Previous studies using Q-methodology have demonstrated that even small samples can reveal meaningful insights into subjective experiences [36]. Additionally, while the sample was not formally stratified by income or education level, it included participants from diverse socio-economic backgrounds, ranging from lower-class to upper-middle-class income groups. This range allowed for a meaningful examination of how financial and educational disparities shape CI decision-making. Future research could build on these findings by expanding the sample size and further stratifying participants based on income, education, and access to healthcare services.

Second, guided responses in the Q-methodology raise a weakness. The predefined Q-set statements may have influenced participants’ responses, potentially limiting the emergence of unanticipated influencing factors. Future research should consider using more open-ended or participant-driven approaches. Third, there were language and literacy constraints. The study required English literacy, potentially excluding non-English-speaking parents. This limitation may have narrowed the range of perspectives, particularly those from linguistically diverse or underrepresented communities. Fourth, is the potential underrepresentation of parents from rural areas. While recruitment was open to all South African parents of DHH children, most respondents were from urban or peri-urban backgrounds, likely due to the digital nature of recruitment and the concentration of CI services in metropolitan regions. Future studies should employ targeted recruitment strategies, such as direct outreach through rural healthcare centres and community networks, to ensure a more balanced representation of rural parental perspectives on CI decision-making. Furthermore, while online recruitment enabled broader outreach, it may have favoured parents with internet access and digital literacy, inadvertently excluding those from lower-income or resource-limited settings. Although efforts were made to recruit participants via healthcare facilities and non-digital channels, this study may still underrepresent parents who lack internet access or are not actively engaged with audiology services. Future research should explore alternative recruitment strategies, such as in-person data collection through rural clinics and community centres, to ensure a more inclusive sample. Fifth, this study relied on self-reported data, which introduces the possibility of response bias. Participants may have overestimated or underestimated certain factors, such as their awareness of CI benefits and risks, financial capabilities, or perceptions of stigma. Additionally, social desirability bias may have influenced responses, particularly regarding attitudes toward Deaf culture and sign language use. Future studies should consider using multiple data sources, including observational methods, longitudinal designs, or validated measures, to mitigate these biases and provide a more objective representation of parental decision-making. Lastly, the sample was not stratified by income level or cultural background, despite the study’s focus on socio-economic and cultural influences. However, the sample still included participants across different income groups and educational levels, capturing a broad socio-economic spectrum. The diversity in financial standing influenced parents’ reported concerns about CI affordability, post-implantation rehabilitation, and public vs. private healthcare access. While stratification could have provided more targeted subgroup comparisons, the emerging themes already reflect socio-economic variation in how parents approach CI decision-making. Future studies should incorporate larger, stratified samples to further investigate how socio-economic status and cultural identity impact parental choices.

## 5. Conclusions

This study provides a nuanced understanding of how socio-economic constraints, cultural perceptions, and healthcare access shape parental decision-making regarding CIs in South Africa. Findings indicate that parents overwhelmingly recognize the potential benefits of CIs, particularly for speech development, but many are hindered by financial barriers, stigma, and inconsistent access to information. The critical role of healthcare professionals in guiding parental decisions was emphasized, although some parents expressed concerns about the transparency and completeness of medical information regarding CIs. Given these findings, several key implications for policy, healthcare practice, and community engagement emerge.

Firstly, public funding and public healthcare support for cochlear implantation and post-implantation rehabilitation should be strengthened and expanded, ensuring that CI programmes are accessible. However, implementing these recommendations in South Africa’s resource-constrained healthcare system poses significant financial and logistical challenges. Limited government budgets and competing healthcare priorities make large-scale CI funding difficult. Potential funding solutions include public–private partnerships, integration of CI services into national health insurance (NHI) frameworks, and collaborations with international donors or hearing health non-governmental organisations (NGOs). Policymakers must balance cost-effectiveness with equitable access, ensuring that CI programmes do not divert critical resources from other essential healthcare services. Future research should explore sustainable funding models and scalable awareness strategies that align with existing public health priorities. Secondly, parental counselling and informed decision-making should be enhanced. Standardized, culturally appropriate CI counselling protocols for audiologists and healthcare providers should be developed to ensure comprehensive, unbiased information is provided. Decision aids (e.g., multilingual brochures, online resources) should be provided to help parents navigate CI options and expectations realistically. Moreover, pre- and post-implantation parental support groups should be offered, allowing parents to connect, share experiences, and receive guidance. Thirdly, stigma and cultural perceptions of CIs should be addressed. National awareness campaigns aimed at reducing stigma around hearing loss and assistive devices should be implemented. Mitigating stigma requires targeted public awareness campaigns, integration of Deaf community perspectives in CI education, and increased representation of CI users in media and advocacy efforts. In South Africa, initiatives such as the promotion of SASL in schools have improved attitudes toward Deaf identity, suggesting that similar inclusive approaches could reduce misconceptions about CIs. More recently, having the 2025 Miss South Africa (Mia le Roux) being a DHH individual with a CI has had a significant impact on mitigating stigma. Future efforts should focus on community-driven education to promote greater acceptance of hearing assistive devices, such as having traditional leaders, community influencers, and educators engaging in promoting positive attitudes toward CIs within different cultural contexts. Moreover, Deaf culture and CI awareness should be incorporated into school curriculums to normalize hearing assistive technology from a young age. Fourthly, education and communication access should be expanded for DHH children. Bilingual education models should be encouraged where ASL and spoken language are taught in parallel, ensuring that DHH children have multiple communication options. Training for teachers in mainstream schools to accommodate children with CIs and other hearing assistive devices should be increased, reducing exclusion in classroom settings. Lastly, there are future research and policy development implications raised. As CI technology continues to advance, it is critical that healthcare systems, policymakers, and communities work collaboratively to ensure equitable access.

## Figures and Tables

**Table 1:** Demographic profile of participants (n = 9).

Category	Subcategory	Number of Participants (n = 9)
School Attendance	Attending a school for the Deaf	2
	Not attending a school for the Deaf	7
Use of Hearing Assistive Devices	Use hearing aids	7
	Consistently use hearing aids	4
	Do not use hearing aids	2
Modes of Communication	Speech	3
	Gestures	3
	Total communication	2
	Sign language	1
Income Range	Lower Class (ZAR 3500–ZAR 8000)	1
	Lower-Middle Class (ZAR 8000–ZAR 22,000)	4
	Middle Class (ZAR 22,000–ZAR 40,000)	2
	Upper-Middle Class (ZAR 40,000–ZAR 75,000)	2
Educational Qualification	Grade 9	1
	High School Diploma	2
	Bachelor’s Degree	6

**Table 2:** Correlation matrix of survey results (n = 9).

Respondent	P1	P2	P3	P4	P5	P6	P7	P8	P9
P1	100	−23	−20	−20	21	41	16	25	38
P2	−23	100	56	5	−42	−25	−16	−39	−28
P3	−20	56	100	25	−32	1	−15	−3	−8
P4	−20	5	25	100	13	−5	−34	−9	5
P5	21	−42	−32	13	100	18	9	22	−19
P6	41	−25	1	−5	18	100	19	12	4
P7	16	−16	−15	−34	9	19	100	37	23
P8	25	−39	−3	−9	22	12	37	100	35
P9	38	−28	−8	5	−19	4	23	35	100

## Data Availability

The datasets used and/or analyzed during the current study are available from the corresponding author on reasonable request.

## Appendix A

Survey

Name (fake name to be used in report): _____________________ Date: ___________

1. Which school/institution does your child attend? ______________________________
2. Does your child use any Hearing Assistive Devices? YES/NO
3. If yes, which device(s)? _________________________________________________
4. How does your child communicate with you and others? (e.g., speech, sign language, gestures, writing, a combination, etc.)

____________________________________________________________________

5. How do you usually communicate with people other than your child?

_____________________________________________________________________

6. Please rate the following statements according to whether you (1) Strongly agree, (2) Agree, (3) Disagree or (4) Strongly disagree:

	**Strongly** **Agree**	**Agree**	**Disagree**	**Strongly** **Disagree**
	**1**	**2**	**3**	**4**
It is unclear whether spoken or sign language is the better option for Deaf/hard-of-hearing children.				
Cochlear implant surgery is unaffordable for most South Africans.				
There are not enough Deaf schools in South Africa where Deaf/hard-of-hearing children can learn and use sign language.				
Being able to use both a sign language and a spoken language can be compared to being able to speak both English and Zulu.				
If a Deaf/hard-of-hearing child can learn to speak, they always should.				
It is not necessary for a Deaf/hard-of-hearing child to learn how to speak.				
A Deaf/hard-of-hearing child should learn a sign language and a spoken language at the same time.				
It is not effective for a Deaf/hard-of-hearing child to learn a sign language and a spoken language at the same time.				
Cochlear implant surgery is always in the best interest of the child.				
Cochlear implant surgery comes with many risks.				
In South Africa, people with cochlear implants are discriminated against.				
South Africa is generally accepting of the Deaf community and people who use sign language.				
Deaf children can effectively learn spoken language.				
Deaf/hard-of-hearing children cannot learn to speak as well as hearing children.				
Little children will adapt quickly to cochlear implants.				
Cochlear implants are uncomfortable for little children.				
Sign language helps to preserve Deaf culture.				
A Deaf/hard-of-hearing person can learn sign language at any age.				
Sign languages will be used more and more in the future.				
People with cochlear implants are still a part of the Deaf community.				
Academic success is not affected by how a child communicates.				
Cochlear implants improve the overall well-being of a Deaf/hard-of-hearing child.				
Sign language allows a Deaf/hard-of-hearing child to communicate more freely.				
Cochlear implant surgery is not as risky as some people make it out to be.				
In South Africa, people who do not use spoken language are discriminated against.				
A Deaf/hard-of-hearing child having a cochlear implant does not mean that they will develop good speech.				
It is important for people living in South Africa to be able to speak English.				

7. What were the key reasons behind your agreement/disagreement with the statements in the survey?

____________________________________________________________________

8. Do you think your viewpoint is common or do you feel that you are in a minority who feel this way? Kindly elaborate.

____________________________________________________________________

9. Would you say that your agreement/disagreement with the statements was influenced by outside factors (e.g., conversations with medical practitioners, conversations with other parents or loved ones, online resources, the media, etc.)? If yes, what were the main external factors?

____________________________________________________________________

10. Would you be willing to participate in an interview about this topic? YES/NO

## Appendix B

Interview Guide

What statements in the survey stood out to you and why?
Did you receive a lot of conflicting opinions about how your child should communicate?
Who were the primary people you turned to for advice/counsel when making a decision about how your child should communicate?
What were the main resources (physical or digital) that you consulted for advice about how your child should communicate?
Do you consider the topic of cochlear implantation to be controversial/sensitive? Why/Why not?
Did you feel a lot of pressure to do certain things (such as pressure to choose a particular communication mode or to implant/not implant your child with a CI?)
What advice do you think was the most valuable or eye-opening to you when making decisions concerning communication mode?
